# CRISPR-based genetic tools for the study of host-microbe interactions

**DOI:** 10.1128/iai.00510-24

**Published:** 2025-08-04

**Authors:** Martin Echavarria Galindo, Yong Lai

**Affiliations:** 1 Department of Chemical and Biological Engineering, Hong Kong University of Science and Technology, Clear Water Bay 567841 , Kowloon, Hong Kong SAR, China; University of California Merced, Merced, California, USA

**Keywords:** CRISPR, host-microbe interactions, genetic tools

## Abstract

CRISPR-based genetic tools have revolutionized our ability to interrogate and manipulate genes. These tools can be applied to both host and microbial cells, and their use can enhance our understanding of the dynamic nature of host-microbe interactions by uncovering their genetic underpinnings. As reviewed here, CRISPR-based tools are being used to explore the microbiome in an efficient, accurate, and high-throughput manner. By employing CRISPR screens, targeted genome editing, and recording systems to the study of host cells and microorganisms, we can gain critical insights into host defense mechanisms, potential vulnerabilities, and microbial pathogenesis, as well as essential or condition-specific genes involved in host-microbe interactions. Additionally, CRISPR-based genetic tools are being used in animal models to study host-microbe interactions *in vivo*. Recent advancements in CRISPR-derived technology can be combined with emerging techniques, such as single-cell RNA sequencing, to examine the complex interactions between hosts and microbes, shedding light on the role of the microbiome in health and disease. This review aims to provide a comprehensive overview of how these cutting-edge genetic tools are being used to investigate host-microbial systems, as well as their current limitations. Current research is likely to yield even more advanced genetic toolkits than those presently available, and these can serve researchers in identifying and exploring new therapeutic targets for diseases related to host-microbe interactions.

## INTRODUCTION

The advent of high-throughput sequencing technologies, in conjunction with advanced metagenomic analyses, has significantly advanced our understanding of what is often referred to as the “human microbiota dark matter.” This term encapsulates the vast diversity of commensal microorganisms that inhabit the skin and mucosal tissues throughout the human body, i.e., the human microbiota, of which the gut microbiota is the most extensively characterized component (1). Current estimates suggest that the human microbiota consists of over 100 trillion microbial cells (2). Until recently, the microbiota was largely overlooked; it is now thought to play a critical role in human health. Characterizing these remarkably diverse microbial communities and their interactions with their hosts is essential to fully appreciate their implications for health and disease, as well as to unlock potential therapeutic avenues.

The composition of the microbiota is highly variable, as are its effects on various human populations (3). Heterogeneity in the gut microbiome is influenced by diet (4), age (5), host genetics (6), and environmental and geographical factors (7). Despite this variability, certain functional attributes, core pathways, and dynamics of the gut microbiome remain relatively consistent across human populations (8).

The gut microbiota contributes to human health by supporting host metabolism. These organisms facilitate the breakdown of complex carbohydrates and other dietary components and synthesize several metabolites essential for human health (9), such as short-chain fatty acids (SCFAs), which have been implicated in the modulation and maturation of the host immune system, among other functions. The SCFA butyrate promotes the differentiation of regulatory T-cells, which are vital for maintaining immune homeostasis (10). SCFAs facilitate the release of cytokines and chemokines, which are involved in the immune response (11). Moreover, SCFAs are instrumental in preventing gut inflammation, enhancing gut barrier integrity, and regulating host energy intake (12).

The interactions between microbiota and host have also been implicated in the pathogenesis of inflammatory bowel disease (IBD) (13), diabetes (14), psychiatric disorders (15), and colorectal cancer (16). Although the precise mechanisms by which the microbiota influences these conditions remain incompletely understood, dysbiosis—an imbalance in the composition of microbial communities, particularly within the gut—may play a significant role in disease onset and progression (17). The integral role of the gut microbiota in maintaining overall health points to the importance of developing new tools to elucidate microbe-host interactions, which could lead to therapeutic strategies for microbiota-related dysbiosis.

### Traditional methods to study host-microbe interactions

**Fig 1:**
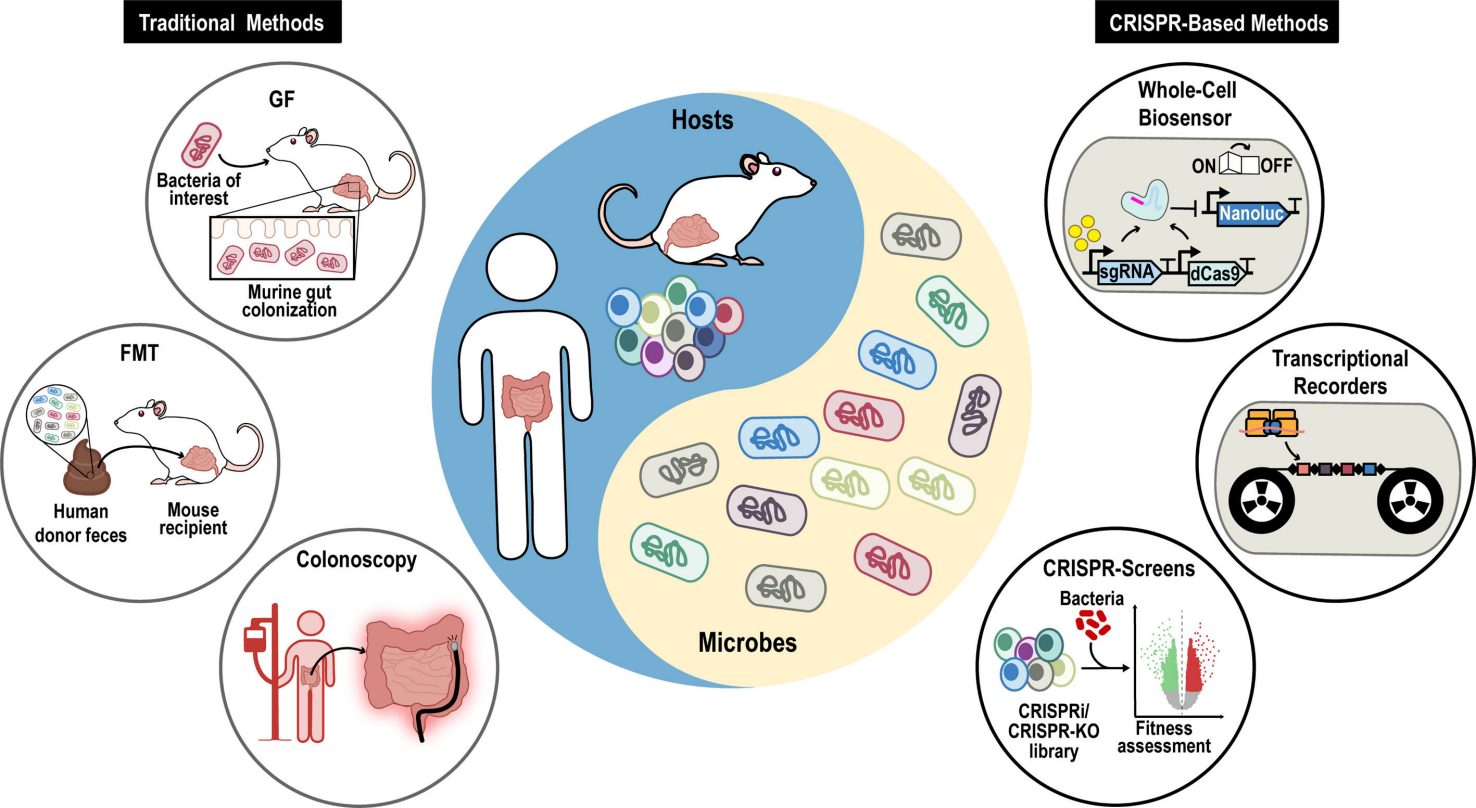
Overview of traditional and CRISPR-based methods for studying host-microbe interactions. Traditional methods (left) include germ-free (GF) mouse models, fecal microbiota transplantation (FMT), and colonoscopy. CRISPR-based methods (right) feature the design of whole-cell biosensors, transcriptional recorders, and CRISPR screens for gene characterization.

The study of host-microbe interactions (Fig. 1, center panel) initially focused on identifying pathogenic bacteria responsible for specific diseases. According to Koch’s postulates, which offer a systematic approach to linking microbes to diseases, a microorganism must be absent in all healthy individuals and present in all diseased patients to be considered a pathogen, as well as being able to cause disease once it is introduced into a healthy host (1). The discovery of *Helicobacter pylori* as a causative agent of chronic gastritis exemplifies how Koch’s postulates have facilitated the identification of pathogenic microorganisms (18).

More recently, causal relationships between specific microbiota members and host phenotypes have been explored with germ-free (GF) animal models (Fig. 1, traditional methods). In these models, mice are colonized with a single bacterial species or an artificial microbial community, and researchers assess the resulting phenotypic changes. This method significantly advanced early research on IBD by identifying bacteria, such as *Escherichia coli* Nissle 1917 (19), *Bacteroides vulgatus* (20), and *Klebsiella pneumoniae* (21), that modulate inflammation in colitis mouse models. However, GF models do not accurately replicate the complexity of interactions within the native microbiota or of potential higher-order interactions among microbial species that contribute to specific host phenotypes. To address these complexities, alternative methods, such as fecal microbiota transplantation (FMT), have been developed. One use of FMT to assess these interactions resulted in the discovery that transferring fecal microbiota from Crohn’s disease patients to GF mice induced colonic inflammation (22). Another example was the discovery of the role that the microbiota may play in obesity: transplanting the uncultured human fecal microbiota from each member of twin pairs discordant for obesity into separate groups of GF mice led to an increase in adipose mass and in the production of obesity-associated metabolites like branched-chain amino acids in mice that had received obese-donor microbiota but not in mice that had received lean-donor microbiota (23). These two examples demonstrate the usefulness of FMT for characterizing the role of gut microbiota in host phenotypes.

Another traditional method is colonoscopy, which allows direct visualization of the bowel and reveals structural changes in the host tissue associated with microbiota alterations. However, this technique is invasive and, because it occurs at a single point in time, may not capture specific details regarding the dynamic behavior of the microbiota.

Recent research has increasingly focused on multiomics approaches to study the dynamic and multifactorial nature of host-microbe interactions. Multiomics is typically integrated with high-throughput next-generation sequencing (NGS) data to identify those microbial species and their associated products that are enriched or depleted under disease conditions (24). This strategy has been used to assess community-level dysbiosis in microbiome-related diseases by identifying, as disease biomarkers, changes in the microbiota and in metabolite composition. Hypotheses about the role of uncultured or poorly characterized bacterial species in disease onset have also been generated based on multiomics, though such approaches generally reveal correlations rather than causation (25). Mechanistic, gene-centered research is needed to test hypotheses derived from multiomics data. By elucidating the molecular mechanisms underlying host-microbiota interactions, such research could clarify the role of microbial metabolic pathways in human health and illuminate how their dysregulation may contribute to disease.

Genome editing tools based on clustered regularly interspaced short palindromic repeats (CRISPR) have enabled precise and targeted gene editing across a wide range of species. CRISPR is not a single technology but, rather, an array of methods for genomic interrogation and manipulation. CRISPR is being applied to the genomes of microorganisms and host cells to probe host-microbe interactions at the genetic level and to mechanistically test relevant hypotheses (Fig. 1, CRISPR-based methods). This review describes current and future applications of CRISPR technologies to these interactions.

## OVERVIEW OF CRISPR TECHNOLOGIES

### CRISPR-based tools for gene editing

The CRISPR-Cas system is a groundbreaking, versatile gene-editing technology, originally derived from the adaptive immune mechanisms of bacteria and archaea. In these microorganisms, CRISPR-Cas provides defense against phage infections (26). The version of this system most widely employed in research and biotechnology is CRISPR-Cas9, which utilizes the Cas9 nuclease from *Streptococcus pyogenes*. In this system, an engineered single-guide RNA (sgRNA) directs the Cas9 nuclease to its complementary DNA sequence (i.e., a specific location in the DNA), where it binds adjacent to a critical short sequence, the protospacer-adjacent motif (PAM) (24), resulting in the targeted cleavage of the double-stranded DNA. The PAM sequence is essential for target binding and activating the nuclease activity of Cas9 (27), facilitating the double-stranded break at the desired site. This precise targeting capability provides unprecedented opportunities for genome modification, functional genomics studies, and routes to novel therapies. For the development of this technology, Jennifer Doudna and Emmanuelle Charpentier were awarded the 2020 Nobel Prize in Chemistry.

Because CRISPR-Cas9 can induce double-stranded breaks in DNA at specified positions, it can be used to precisely target specific bacteria, such as antibiotic-resistant or pathogenic strains. By designing sgRNAs that target genes unique to the strains of interest, researchers can effectively direct Cas9. Double-stranded breaks in plasmid DNA can lead to plasmid loss, whereas when such breaks occur in genomic sequences, the result may be DNA degradation and subsequent cell death (28). Because the non-homologous end-joining repair mechanism is less prevalent in prokaryotes than in eukaryotes, the likelihood of repair is reduced, and the lethality of the breaks is enhanced (29). Additionally, the CRISPR-Cas9 system can facilitate targeted gene insertion if a repair template is provided; in this case, homology-directed repair repairs the double-stranded breaks (30).

There are other methods of targeted genetic modification besides traditional CRISPR-Cas9 systems. Base editing extends the capabilities of the conventional CRISPR-Cas9 approach, allowing for precise and efficient genetic alterations without the need to induce double-stranded breaks, by using either a Cas9-nickase or “dead Cas” (dCas), a catalytically inactive version of the Cas enzyme, both of which are guided by sgRNAs to specific DNA sites. One prominent type of base-editing tool is the cytosine base editor, which fuses the Cas9-nickase/dCas protein with a cytosine deaminase enzyme, converting specific cytosines to uracils at designated sites within the DNA sequence (31). During DNA replication, the uracils are read as thymines by DNA polymerase, resulting in a C-to-T transition (31). Another variant of base editing is the adenine base editor, which involves the fusion of the Cas9-nickase or dCas protein with a TadA* deoxyadenosine deaminase, thus converting specific adenosines into inosines, which are interpreted by the cellular replication machinery as guanines, leading to an A-to-G transition (32). Base-editing tools have been successfully implemented in multiple bacterial organisms, including, notably, *E. coli* (33), *K. pneumoniae* (34), and *Bacillus subtilis* (35).

CRISPR-based DNA prime-editing tools can introduce a wide array of genetic alterations (insertions, deletions, and all 12 possible base-to-base conversions) without necessitating double-stranded breaks. Prime editors typically comprise a Cas9-nickase enzyme fused to a modified reverse transcriptase. This complex is directed to the target site within the DNA sequence of interest by a prime-editing guide RNA (pegRNA), which both guides the prime editor and encodes the desired sequence modification at its 3′ end. Upon binding to the target site, the Cas9-nickase creates a single-stranded nick, and the nicked DNA strand serves as a primer for the reverse transcriptase to synthesize the sequence encoded by the 3′ end of the pegRNA and incorporate it into the DNA (36). This process thus introduces the intended genetic alteration at the desired position. Prime editing tools have been successfully implemented in eukaryotes such as human (37) or mice cells (38), but their implementation in bacteria has been somewhat limited due to their low editing efficiency in these organisms (39). Nevertheless, some examples of bacteria for which prime editing has been effectively applied are *E. coli* (39), *Streptococcus pneumoniae* (40), and *Mycobacterium smegmatis* (41).

One of the main limitations of CRISPR-based gene editing tools is the risk of off-target effects. These effects can be attributed to several factors, primarily the use of Cas9 variants with relatively high incidences of off-target binding (42) and incorrect sgRNA design (42, 43), highlighting the need to optimize these two factors for CRISPR-based experiments. Another major problem is the PAM recognition constraint of Cas9, which restricts the range of targetable sequences (44). Although engineered PAM-flexible Cas9 variants have been developed, studies have demonstrated a trade-off between PAM flexibility and targeting specificity, often resulting in increased off-target activity (45). Additional challenges include the potential cytotoxicity of Cas9 nucleases in certain host systems, delivery obstacles for different organisms, and host-specific factors that may influence editing efficiency and outcomes.

### CRISPRi/CRISPRa for tight control of gene expression

Two techniques for regulating CRISPR-mediated gene expression have advanced genetic engineering: CRISPR interference (CRISPRi) and CRISPR activation (CRISPRa).

In CRISPRi, the dCas protein is guided by an sgRNA to target RNA polymerase binding sites within promoter sequences, thereby blocking transcription initiation (46). Alternatively, sgRNAs can target the coding strand of a gene to obstruct transcription elongation (47). This steric hindrance mechanism offers a significant advantage over traditional repressor methods, as the repressive action of CRISPRi depends solely on the binding of sgRNA to its target sequence, independent of the host’s cellular machinery (48, 49).

CRISPRi systems have now been implemented in over 80 different bacterial species (50), from commonly used model laboratory bacterial species such as *E. coli* (46) and *B. subtilis* (51) to pathogenic bacteria such as *Mycobacterium tuberculosis* (52–54), *Listeria monocytogenes* (55), *S. pneumoniae* (56), and *Enterococcus faecalis* (57). Apart from bacteria, CRISPRi systems have been implemented in mammalian cells (58), in fungal species such as *Saccharomyces cerevisiae* (59), and in archaea such as *Haloferax volcanii* (60).

CRISPRi can also be used in a high-throughput manner to characterize gene function in a method often referred to as CRISPR screens. These high-throughput screens can be carried out either in a pooled manner (i.e., pooled CRISPR screens) or in an ordered or arrayed manner (i.e., arrayed CRISPR screens). In pooled screens, the sgRNA library is introduced in bulk into a cell population, and the effect of the perturbations is assessed by measuring the enrichment or depletion of each of the library members in the pool of cells after the challenge. In arrayed screens, a cell population is physically separated (subpopulations are placed in different wells of a multiwell plate), and each subpopulation receives one sgRNA (i.e., each well contains a population carrying the same mutation).

CRISPRi screens are simpler than traditional methods, such as transposon insertion sequencing (Tn-seq), as they require only the expression of the CRISPR-Cas system and the sgRNA library to silence gene expression. The elements involved are easy to synthesize and amenable to high-throughput sequencing. CRISPRi screens are also modular as they require only two main components, i.e., the dCas protein and the sgRNA library, the latter of which can be customized depending on the biological question. In these screens, CRISPR-based silencing depends only on the specific sgRNA used to target the locus of interest, so a small library of sgRNAs can be used to analyze specific genes. In contrast, Tn-seq involves the random insertion of transposon elements. Because CRISPR-based silencing can be designed to be inducible and dose-dependent, it can be used to study essential genes (i.e., genes required for bacterial survival): unlike Tn-seq, which leads to the total knockdown of the affected genes, CRISPR-based silencing can partially knock down gene expression (61). However, as non-model bacteria may respond differently to the same dCas9 protein, CRISPR-screening protocols should be optimized for each bacterial species. Optimization should address construct design and expression, choice of delivery system, and the design of the sgRNA and dCas9 variants of choice (62). Furthermore, the sgRNA should be designed to avoid possible off-target effects such as aberrant phenotypes, which can lead to improper fitness measurements.

Conversely, CRISPRa promotes the expression of target genes by emulating the function of transcription factors. With CRISPRa, the dCas protein, which acts as the DNA-binding domain, is fused to a transcriptional activation domain, which interacts with the transcriptional machinery, thereby promoting gene expression. CRISPRa technologies have been developed for model bacteria such as *E. coli* (56), non-model bacteria such as the pathogenic *Salmonella enterica* (63), and multiple fungal pathogens such as *Candida albicans* (64) and *Nakaseomyces glabrata* (65).

The choice of transcriptional activation domain is critical to the efficacy with which CRISPRa upregulates targeted gene expression. Various transcriptional activation proteins have been identified, each differing in their host range and transcriptional activation capabilities. The AsiA protein, for example, can induce gene expression across multiple promoters and bacterial hosts (63). CRISPRa has also been implemented in mammalian cells in the form of CRISPR-based artificial transcription factors (CRISPR-TF), by fusing the sgRNA-dCas9 complex with transcription activation proteins, such as VP64 (66), VPR (VP64-p65-Rta) (67), SunTag (68), or SAM (69). The development of CRISPR-TFs has facilitated the creation of CRISPR-TF-compatible promoters. These promoters, which provide tight transcriptional control, make it possible to design complex transcription systems with orthogonal regulation in mammalian cells by modulating the sgRNA sequence, the number of binding sites within the promoter, and the specific activator proteins utilized (70).

## CRISPR-BASED INTERROGATION AND ENGINEERING APPROACHES IN HOST-MICROBE RESEARCH

This section explores the application of diverse CRISPR-based tools for characterizing host-microbe interactions, encompassing both host and microbial perspectives. Key studies illustrating these applications are summarized in Table 1.

**TABLE 1:** Key studies using CRISPR-based tools to investigate host-microbe interactions

Purpose	Target organism	Assay type	Area of investigation or result	Reference
Whole-cell biosensor design	*Bacteroides thetaiotaomicron*	CRISPRi-based genetic circuit	Repression of reporter gene *in vivo* under Isopropyl β-d-1-thiogalactopyranoside (IPTG) conditions	(49)
	*Vibrio cholerae*	CRISPRi-based genetic inverter	Expression of a repressed reporter gene after sensing of signaling molecule	(71)
Recording of transcriptomic signals	*E. coli*	Record-seq	*In vivo* recording of transcriptomic changes induced by host dietary changes or changes in the gut microbial community	(72)
Discovery of key microbial factors	*E. faecalis*	Target-specific CRISPRi	Reduction of biofilm formation after CRISPRi silencing of the biofilm-forming operon	(57)
	*S. pyogenes*	Target-specific CRISPRi	Decrease in bacterial virulence in mouse *in vivo* models following CRISPRi silencing of the FtsZ gene	(73)
	*Streptococcus mutans*	Target-specific CRISPRi	Decreased virulence in a waxworm *in vivo* model following CRISPRi silencing of the rhamnose-glucose polysaccharide synthesis	(74)
	*M. tuberculosis*	Genome-wide CRISPR screens	Susceptibility of *M. tuberculosis* to inhibition of redox homeostasis, amino acid metabolism, and protein synthesis pathways, revealed by genome-wide screening	(54)
	*S. pneumoniae*	Genome-wide CRISPR screens	Key role of genes for capsule production and adenylsuccinate synthetase in bacterial growth during Influenza A co-infection in mouse model, elucidated by *in vivo* genome-wide screening	(75)
Discovery of host factors	THP-1 human monocytic cell line	Genome-wide CRISPR screens	Improved phagocytic cell survival in *Mycobacteria* infection following inhibition of type I interferon and aryl hydrocarbon receptor signaling pathways, shown by genome-wide screening	(76)
	HT-29 human intestinal epithelial cell line	Genome-wide CRISPR screens	Knockdown of cell surface sulfation and protein fucosylation confers protection against type III secretion system (T3SS)-mediated cytotoxicity in host cells during *Vibrio parahaemolyticus* infection, revealed by genome-wide CRISPR screens	(77)

### CRISPR-based tools for the design of biosensors and whole-cell recorders

Current surgical methods for assessing gut health are invasive, complex, and expensive. In contrast, bacterial strains can be engineered as whole-cell biosensors to sense and respond to environmental cues (Fig. 1). These biosensors are non-invasive and yet can access regions of the gut that traditional methods cannot easily reach, interact directly with the host’s microbiota, and detect transient biomarker metabolites. Furthermore, the data output from the biosensors can be conveniently collected from fecal samples, making the process less intrusive for patients.

Biosensors can also be engineered to deliver active therapeutic molecules or agents, *in vivo*, for various diseases. Notable examples of biosensors engineered for the delivery of such therapeutic payloads include the *E. coli* (19) strain, which has been engineered to express phenylalanine-degrading enzymes for the treatment of phenylketonuria (78); *Lactococcus lactis*, which has been engineered to secrete proinsulin autoantigen and IL-10 to treat type-1 diabetes (79); and *Bacteroides ovatus*, which has been engineered to secrete the transforming growth factor-β (TGF-β) to treat colitis (80). Genetically engineered probiotic biosensors can be integrated with microelectronics for real-time non-invasive disease monitoring in the gastrointestinal tract of porcine models (81, 82).

This section will explore the application of CRISPR to the design of state-of-the-art biosensors and whole-cell recorders and their use for assessing gut health.

#### CRISPR-based tools for biosensor design

Biosensor design hinges on the ability of engineered bacteria to detect an external stimulus of interest, such as a small molecule metabolite that serves as a biomarker for a particular condition. Once the bacteria detect the stimulus, they then process it intracellularly, leading to a downstream response, such as the production of a specific protein or the recording of cellular activity (83). The high binding specificity of CRISPR-based tools and their ability to furnish precise control over gene expression make them exceptionally well-suited for such applications.

**Fig 2:**
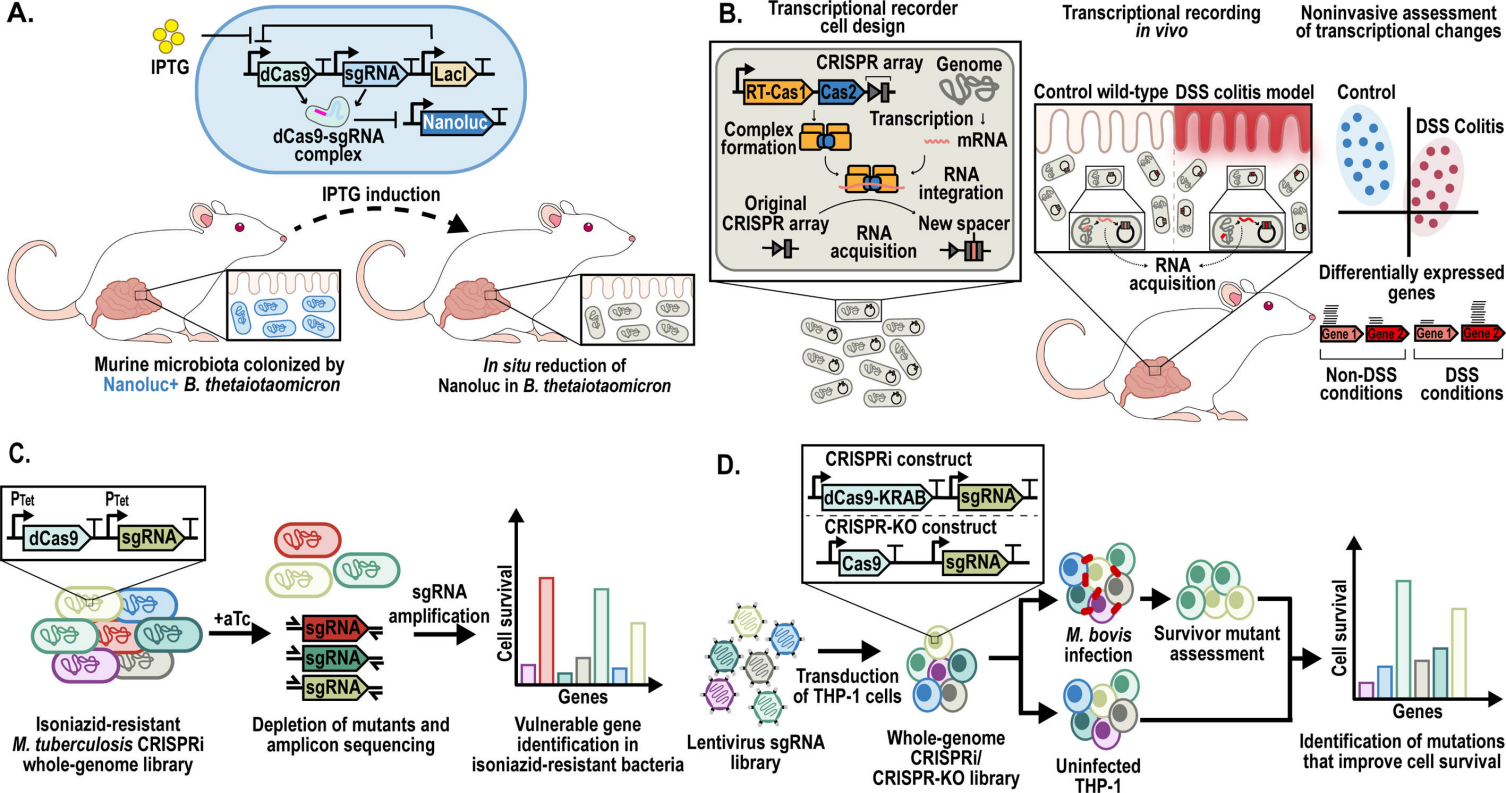
Application of CRISPR methods for studying host-microbe interactions. (**A**) CRISPRi-based genetic circuits have been designed for precise, stimulus-responsive gene inhibition in *B. thetaiotaomicron* in the murine gut (49). (**B**) Record-seq, a CRISPR-based molecular recording method, has been used to design transcriptional recorders that detect diet- and inflammation-specific transcription signals *in vivo* (72). (**C**) CRISPRi genome-wide screening in antibiotic-resistant *M. tuberculosis* revealed novel vulnerabilities (54). (**D**) Genes improving the survival of THP-1 human phagocytic cells infected by *Mycobacterium bovis* were identified by whole-genome CRISPR-KO/CRISPRi screening (76). DSS, dextran sodium sulfate.

CRISPR systems can be used to control gene expression within complex genetic circuits in *in vivo* environments, as has been previously attempted in *Bacteroides thetaiotaomicron*, which is one of the most prevalent bacteria in the human gut and is notable for its capacity for long-term colonization, as evidenced by its low clearance rate in this environment (84). In this study, an IPTG-inducible CRISPRi system was designed to repress a reporter gene (Nanoluc) in *B. thetaiotaomicron* (Fig. 2A and Table 1), enabling time-dependent and highly sensitive detection of gene silencing signals in mice (49). This serves as a proof-of-concept output assay showcasing the potential to couple CRISPRi with sensor modules for the future development of biosensors.

Sophisticated biosensors, built with CRISPR technology, can operate with precision and be adapted to diverse biological settings. CRISPR-based genetic circuits have been utilized to develop biosensors to detect pathogenic bacteria. Notably, a CRISPR-based genetic inverter circuit constructed in *E. coli* leverages the quorum-sensing mechanism of *Vibrio cholerae* for the signaling molecule CAI-1 to induce the expression of green fluorescent protein in a specific and density-dependent manner (Table 1) (71).

Genetic circuits for the controlled and targeted delivery of therapeutic payloads are being integrated into biosensors (see “CRISPR-based tools for the design of biosensors and whole-cell recorders,” above, for representative examples of payloads and their associated disease applications) as the basis of targeted therapies for a wide spectrum of clinical conditions. However, substantial work remains to be accomplished in integrating CRISPR-based tools with diverse genetic circuit toolkits tailored to function in various organisms of interest. In addition to this integration, there is a pressing need to identify biomarker metabolites that are associated with specific clinical conditions. Thus, further work is needed to enhance the versatility of these systems and expand their applicability to a range of biological contexts. The real-time monitoring and modulation of cellular responses can thereby enable effective therapeutic interventions.

#### CRISPR-based tools for recording host-microbe interactions

The CRISPR-mediated adaptive immune response allows bacteria to defend themselves against phage and mobile genetic elements (MGEs). In bacteria, spacer acquisition is critical to this response. During this process, spacers (i.e., short segments of foreign DNA derived from these invaders) are integrated into a specific genomic region, the CRISPR locus. The spacers are flanked by direct repeats, creating a unique genetic signature that facilitates the recognition and neutralization of future threats (85).

Spacer integration is orchestrated by the Cas1-Cas2 protein complex, which functions as an integrase. By inserting newly acquired spacer sequences into the leading region of the CRISPR locus, this complex updates the bacterial immune memory (86). New spacers keep being added to the leader sequence, so researchers can reconstruct the chronological sequence of past infections, revealing the evolutionary history of bacterial interactions with foreign genetic elements (87) and suggesting how CRISPR-based tools might be used to record biological events (Fig. 1).

CRISPR-based recording systems offer a powerful platform for monitoring and interrogating MGEs, including plasmids and viruses, which shape the genetic diversity and functional dynamics of the gut microbiota. These recording systems enable the precise monitoring of MGE transfer between bacterial populations, offering insights into their role in modulating microbiome composition and function. However, over 90% of viral sequences in the human gut are estimated to belong to poorly characterized viruses with minimal homology to existing reference databases, highlighting a significant gap in our understanding of gut viromes (88). Given the importance of these genetic elements, their dynamic behavior and interactions within the gut ecosystem merit investigation. CRISPR-based tools also offer promising avenues for the *in situ* characterization of the dynamic processes that govern horizontal gene transfer, a key mechanism driving the rapid spread of antibiotic resistance, a significant public health threat (89).

The recording *E. coli* strain (EcRec) has been engineered to inducibly overexpress the Cas1-Cas2 complex. This engineered strain can record spacers from transient plasmids that do not replicate within the recipient recording bacteria, phages, and even from complex horizontal gene transfer events, as demonstrated when the recording strain is exposed to the diverse donor populations found in human fecal microbiome samples (90). Furthermore, this versatile strain can be used to detect the transfer dynamics of genetic elements in complex microbial communities, which is difficult to detect with phenotype-based methods.

As noted above, because spacer integration is time-dependent, CRISPR systems can potentially function as molecular recorders. However, this application has been limited, as Cas1-Cas2 systems exclusively integrate DNA-derived spacers (not RNA) and, therefore, cannot capture crucial intracellular information, such as transient transcriptomic changes. This limitation was addressed with the discovery of a reverse transcriptase-containing Cas1 (*FS*RT-Cas1) protein in *Fusicatenibacter saccharivorans* (91). This breakthrough led to the development of Record-seq, which uses the native Cas1 and Cas2 proteins from *F. saccharivorans* (i.e., the *FS*RT-Cas1-Cas2 complex) to incorporate RNA-derived spacers into the CRISPR locus (Fig. 2B and Table 1). This system can effectively record transient transcriptomic changes in *E. coli* that occur in response to a wide range of stimuli; the recording process is dependent on RNA abundance (91).

Record-seq technology presents a groundbreaking approach for the non-invasive, *in situ* recording of transient transcriptomic changes within complex microbial communities such as those in the gut microbiome. Unlike traditional RNA-seq methods, which necessitate direct extraction of bacterial RNA, Record-seq can capture transcriptomic changes without disrupting the microbial environment. Record-seq could record transcriptomic changes *in vivo* in germ-free mouse models; these changes included (1) the upregulation of carbon utilization pathways in response to host dietary changes or to an increase in the complexity of the microbial community, and (2) the upregulation of oxidative stress response genes under conditions of dextran sodium sulfate (DSS)-mediated inflammation (Fig. 2B and Table 1) (72).

The identification of disease- or condition-specific biomarkers is requisite for developing whole-cell biosensors. Record-seq is being used to identify such biomarkers by characterizing spatio-temporal transcriptional changes. However, the broader application of this technology is limited by its relatively low spacer acquisition efficiency, even though this efficiency is somewhat better than that of traditional Cas1-Cas2 methods. This inefficiency necessitates the use of a large population of recorder cells to reliably capture signals of interest (83).

The Record-seq technique offers a broad overview of global transcriptomic changes but has limited ability to capture detailed information about transcriptional changes in specific genes, because spacer acquisition by the *FS*RT-Cas1-Cas2 complex is promiscuous. To address this limitation, Retro-Cascorder was developed (92). With this method, retron reverse transcriptase (Retron-RT) converts RNA elements containing retron recognition elements (retron non-coding RNA, or retron ncRNA) into single-stranded DNA, which is then inserted into the CRISPR locus in a time-dependent manner (92). For the targeted recording of the activity of a specific promoter, a retron ncRNA is first placed downstream of the promoter of interest. Upon expression, these ncRNA-containing transcripts are selectively recorded in the CRISPR locus, providing a precise record of promoter activity. However, optimization is needed to enhance spacer acquisition efficiency, system portability, and time resolution.

CRISPR-mediated spacer acquisition methods have demonstrated promise in recording interactions with MGEs and capturing transient transcriptomic changes in the gut microbiota. Protein engineering is essential to enhance the efficiency of these systems and to adapt them to a variety of bacterial hosts.

### Microbial genomic modifications for the discovery of key microbial factors

Recent advances in metagenomic sequencing and analysis have allowed new insights into the dynamic composition of the human gut microbiota at both the species and strain levels, as well as correlations between different microbial profiles with specific phenotypes. However, the functional roles of individual genes within this complex microbial ecosystem and how they mediate host-microbe interactions are not entirely clear. CRISPR-based functional genomics can illuminate the genetic mechanisms underlying these interactions.

Reverse genetics approaches can be used to elucidate the roles of specific genes and their contributions to particular phenotypes. A target gene is identified, and the phenotype resulting from its knockdown is evaluated. A typical pipeline for single-gene interrogation in complex microbial communities involves manipulating the gene of interest within a particular strain and comparing the resultant microbial composition and host phenotypes to those of a control group (93).

Along with insights gained with reverse genetics, CRISPRi approaches can reveal how specific genes within the gut microbiome, such as those involved in biosynthetic pathways, influence the interactions between a single commensal bacterial strain and its human host. For instance, a CRISPRi system targeting the biofilm synthesis operon in the pathogenic bacterium *E. faecalis* significantly reduced biofilm formation and prevented bacterial attachment, thereby enhancing antibiotic clearance during infection (Table 1) (57). A CRISPRi assay of genes involved in rhamnose-glucose polysaccharide synthesis in *Streptococcus mutans*, a bacterium associated with dental caries, resulted in impaired cell division, abnormal cell morphologies, and decreased virulence in a *Galleria mellonella* waxworm infection model (Table 1) (74). CRISPRi has also been employed to silence the FtsZ gene, which encodes the tubulin-like cell division protein in the pathogenic bacterium *Streptococcus pyogenes*; gene silencing reduced virulence in mouse *in vivo* models, demonstrating that FtsZ is a potential therapeutic target for combating infections (Table 1) (73).

Similarly, CRISPR screens targeting carbohydrate metabolism genes in *M. tuberculosis* have been used to investigate macrophage activation and bacterial clearance mechanisms. These studies have identified cholesterol import and catabolism genes as key for bacterial intramacrophage growth and survival under macrophage activation by all-trans-retinoic acid (53), suggesting that cholesterol starvation may represent a path to macrophage-mediated clearance of *M. tuberculosis* and highlighting its potential as a therapeutic target for tuberculosis. These examples illustrate the utility of CRISPRi in analyzing gene functions and identifying strategies for therapeutic intervention.

While single-gene studies can enhance our understanding of microbial genes and pathways, a broader comprehension of host-microbe interactions often necessitates a forward genetics approach. This approach, which starts with a phenotype of interest and traces back to identify the gene mutations responsible for that phenotype, is effectively implemented through CRISPR screens, in which a library of sgRNAs each targets a different gene, to facilitate high-throughput genetic silencing. The fitness changes induced by these knockdowns can be assessed by measuring the abundance of each sgRNA in the population using NGS. This method allows researchers to identify the genes that are crucial for survival under various conditions by comparing the differential enrichment of genes between the experimental and baseline conditions. CRISPR screens can reveal the genetic determinants of microbial fitness and adaptability, thereby illuminating host-microbe interactions.

Genome-wide CRISPR screens have also proven invaluable for investigating pathogenesis and uncovering potential therapeutic targets. Recently, genome-wide CRISPR screening of an isoniazid-resistant *katG* (a catalase-peroxidase that participates in redox homeostasis) mutant strain of *M. tuberculosis* (Fig. 2C and Table 1) revealed this strain to be especially vulnerable to the inhibition of genes involved in redox homeostasis, amino acid metabolism, and protein synthesis. Chemical inhibitors targeting these vulnerable pathways could potentially be used to eliminate these antibiotic-resistant pathogenic bacteria (54). A similar strategy was used to identify genes involved in lipoprotein transport in *V. cholerae* (94) and in cell wall biosynthesis in *S. pneumoniae* (95) as possible drug targets.

Genome-wide CRISPR screens are also valuable for studying interactions within complex microbial communities, such as host pathways involved in infection and the effects of phage-resistant bacteria. CRISPR screening prior to phage infection identified genes in *E. coli* whose silencing led to the development of a colanic acid capsule, which acted as a physical barrier that prevented phage from interacting with their entry receptors, conferring broad immunity (47). Similarly, dCas12a-mediated whole-genome CRISPR screens have been employed to assess phage gene essentiality during host infection (96).

CRISPR screens can also be employed to identify key genes mediating host-microbe interactions *in vivo*. However, the loss of library members may be substantial, so that the enrichment of library members becomes a random process rather than a true reflection of fitness (75, 97). Additional challenges include host variability and the poorly understood pharmacokinetics of commonly used bacterial small-molecule inducers.

Yet, there have been notable advances. For example, *in vivo* CRISPR screens used to analyze interactions between the Influenza A virus and *S. pneumoniae* in a mouse co-infection model identified several genes as crucial for promoting bacterial growth in these conditions, including those involved in capsule production and adenylsuccinate synthetase, with the latter being critical due to restricted purine availability in the host environment (Table 1) (75). Thus, *in vivo* CRISPR screens can be used to uncover genetic factors critical to host-microbe interactions.

### CRISPR-based methods for dissecting host responses

CRISPR-based gene editing and functional genomic screens have been applied in mammalian cells for dissecting host responses in the context of host-microbe interactions, primarily in *in vitro* studies using cell lines. These techniques are unlikely to identify pathways and genes that enable pathogenic microbes to survive in their hosts. The inherent technical challenges associated with delivery and the *in situ* editing of host cells have limited their application, but CRISPR technologies are starting to be harnessed to elucidate host-microbe interactions in *in vivo* systems.

CRISPR screens have been extensively used to study the main regulators of immune responses in the context of pathogen infection, inflammation, and immuno-oncology (98). The use of genome-wide CRISPR screening to identify host factors that contribute to pathogen susceptibility has facilitated the identification of the type I interferon and aryl hydrocarbon receptor signaling pathways as potential targets for therapeutic inhibitors aimed at enhancing the survival of human phagocytic cells during *Mycobacterium* infections (Fig. 2D and Table 1) (76). CRISPR screens have also provided mechanistic insights into phagocyte-pathogen interactions during *Shigella flexneri* infection; these studies demonstrated that suppressing host genes associated with the Toll-like receptor 1/2 (TLR1/2) signaling pathway and pyruvate catabolism promotes host cell survival, thus providing important insights for developing therapeutic treatments against shigellosis (99).

Similarly, CRISPR screening has identified that knockdown of genes involved in actin dynamics and cholesterol biosynthesis confers resistance to *Salmonella* infection in phagocytic cells (100). Additionally, these screens have revealed that disruption of host cell autophagy reduces the viability of *Brucella*-infected cells, thereby attenuating *Brucella* infection (101).

CRISPR screens have also been used to systematically characterize the cytotoxicity mediated by type III secretion systems (T3SS) and to identify host factors that confer resistance to such virulence mechanisms. A notable example involves the pathogenic bacterium *Vibrio parahaemolyticus*, a leading causative agent of gastroenteritis. This study revealed that host susceptibility to infection is significantly modulated by genes associated with sulfated glycosaminoglycan synthesis, which promotes bacterial adhesion to host cells, as well as by genes involved in surface protein fucosylation, which facilitates translocon insertion and effector protein delivery (Table 1) (77).

Several innovations have enhanced the resolution and minimized the potential noise inherent in high-throughput CRISPR screens, resulting in refined screening pipelines and streamlining data evaluation. One example is Perturb-Seq, which integrates CRISPR screens with subsequent single-cell RNA sequencing of individual library members (102). This combination can be used to investigate the effects of single mutations on single-cell transcriptomic profiles, offering a comprehensive understanding of observed phenotypes and allowing the reconstruction of gene regulatory networks (98).

Although Perturb-Seq has yet to be applied specifically to the study of host-microbe interactions, it has already been used to functionally characterize host factors facilitating severe acute respiratory syndrome coronavirus 2 infection (103), to identify host factors promoting endosymbiosis during *Toxoplasma gondii* infection (104), and to analyze immune cell activation in response to bacterial lipopolysaccharides (105). Currently, the high cost of single-cell RNA sequencing limits the usefulness of Perturb-Seq for screening large numbers of genes. For this reason, single-cell CRISPR screen methods are often employed only as secondary screens, following the identification of genes of interest from bulk CRISPR screens (98).

Cell lines can be used to gain valuable insights into host factors, such as genes that confer immunity, but do not accurately replicate the native conditions of living organisms. Intestinal organoids have been proposed as a more physiologically relevant model for studying host-microbiome interactions, though they are not yet being used extensively. In one study, genome-wide CRISPR screens in human small intestinal organoids were used to identify genes responsible for resistance to the tumor-suppressive effects of TGF-β (106). Despite the need for high transfection rates, the significant costs, and the inherent complexities of organoid construction (107), CRISPR screening in organoids holds promise for investigating the effects of genetic mutations on host-microbe interactions. Perturb-Seq and other single-cell transcriptomic techniques are anticipated to illuminate these effects in organoids and ultimately to contribute to building a comprehensive model of host-microbiome interactions. In addition to the use of CRISPR screens in organoids, integrating CRISPR screens with spatial transcriptomics presents a promising alternative for characterizing host-microbe interactions within the relevant tissue; such integrated screening has been applied to mouse lung cancer models for evaluating the effects of specific gene knockdowns on tumor fitness (108).

CRISPR screens are not the sole method available to explore host-microbe interactions from the host’s perspective. Emerging techniques, such as CRISPR-based recording systems, can offer valuable insights. Whereas Cas1-Cas2 mediated recording systems are limited in their ability to document biologically relevant phenomena, prime-editing tools like DNA Typewriter have demonstrated functionality in mammalian cells, enabling the recording of specific signals (109).

Base-editing tools induce single-base changes at specific loci; *in vitro* testing of these tools in both bacterial and mammalian systems has shown that they capture biologically significant signals. Stimuli-dependent base editing can be achieved by placing the sgRNA under the control of a promoter responsive to the stimulus of interest. This approach has recorded phage infections of bacteria *in vitro* and the activation of the *Wnt* pathway in mammalian cells (110). These tools hold promise for recording biological events in the gut microbiome.

## TARGETED DELIVERY OF CRISPR-BASED GENE EDITING TOOLS TO MICROBIAL COMMUNITIES

Research on the gut microbiome has been impeded by obstacles to both targeted delivery and the *in situ* editing of specific bacterial populations. Genome editing of commensal gut bacteria frequently requires isolating the bacteria of interest, but substantial numbers of commensal gut bacteria cannot easily be cultured under laboratory conditions (111). Innovative techniques are needed to allow researchers to circumvent the culturing step and facilitate direct manipulation within the gut microbiome.

**Fig 3:**
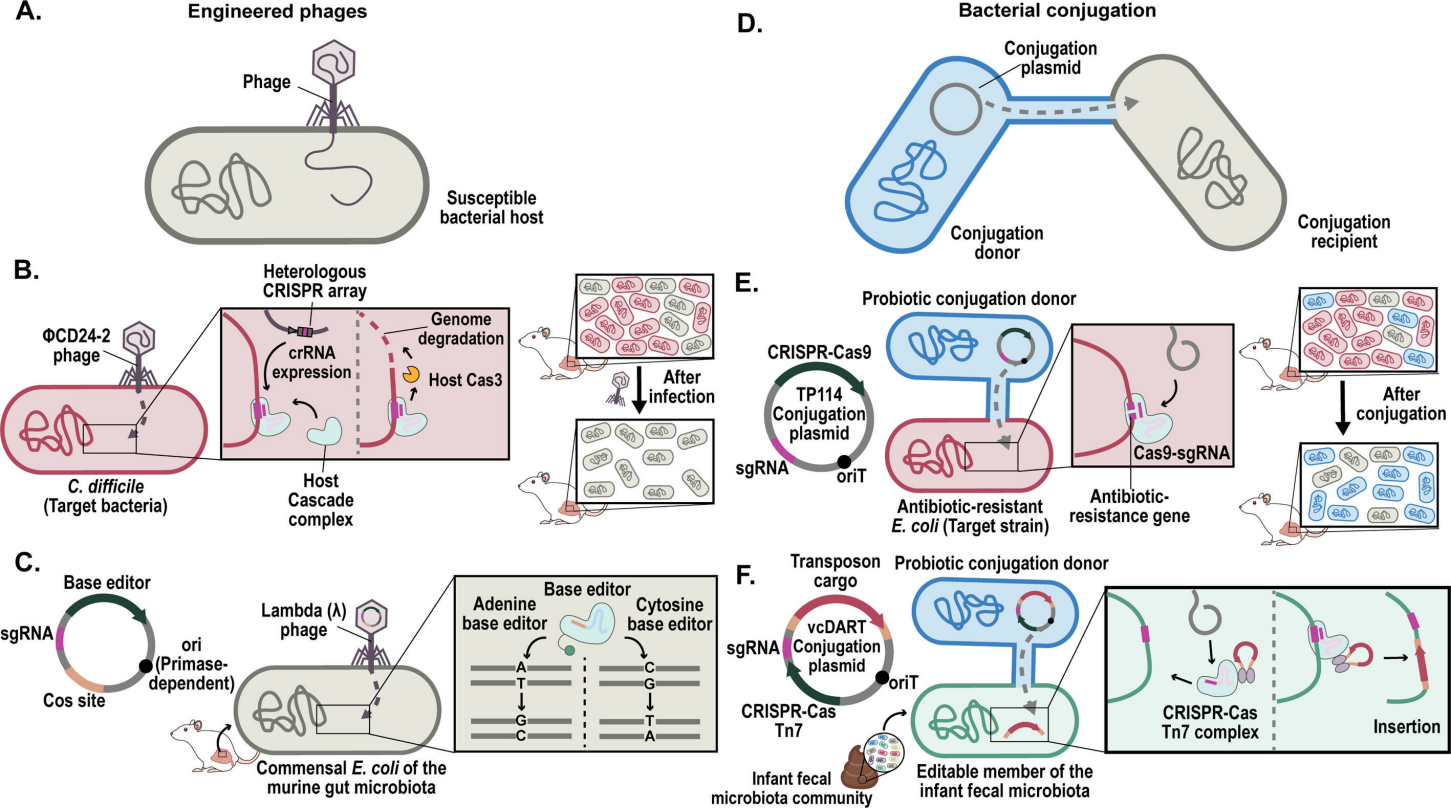
Strategies for the *in situ* delivery of CRISPR tools to complex microbial communities. (**A**) Phages provide highly specific gene delivery, infecting only susceptible bacterial hosts. (**B**) Engineered phages infect *Clostridioides difficile*, depleting these bacteria in the murine gut by inducing genome degradation through a heterologous CRISPR array, with contributions from endogenous Cas3 and Cascade complexes (112). (**C**) Engineered phages deliver base editors into commensal bacteria in the murine gut microbiota, enabling precise *in situ* gene editing (113). (**D**) Bacterial conjugation facilitates low-specificity DNA delivery, wherein donor bacteria transfer a conjugative plasmid containing the desired genetic payload to recipients. (**E**) Probiotic donor bacteria deliver a CRISPR-Cas9 system specifically targeting antibiotic resistance genes, rendering antibiotic-resistant bacterial strains in the murine gut microbiota sensitive to antibiotics (114). (**F**) Probiotic conjugation donors transfer a CRISPR-Cas Tn7 complex, facilitating the insertion of transposons into specific bacteria in an infant’s fecal microbiota (115).

Phage-based systems (Fig. 3A) have emerged as the preferred method for the *in situ* targeted delivery of DNA cargo, such as CRISPR systems, into specific gut bacteria. Phages are highly specific, as particular phages infect only a limited range of bacteria (i.e., their host range) and deliver their cargo efficiently.

Phages have been used to deliver CRISPR systems that deplete antibiotic resistance genes in antibiotic-resistant *Staphylococcus aureus* in *in vivo* mouse skin models (116), as well as targeting *E. coli* (117) and *Clostridioides difficile* (Fig. 3B) (112) within the mouse gut microbiota. Phages have also been employed to deliver CRISPR-based gene editing tools for *in situ* modification; for example, a base editor system targeting commensal *E. coli* strains within the murine microbiota was delivered by engineered phage (Fig. 3C) (113).

Phage-based CRISPR delivery typically involves either temperate phages or phagemids. Temperate phages, which primarily follow a lysogenic life cycle like that of phage Lambda (λ), are preferred for targeted gene editing or gene expression modification within microbial communities, because the CRISPR payload is first inserted into the phage genome and then, upon infection, the phage integrates into the bacterial chromosome, ensuring the maintenance of the CRISPR payload in the bacterial cell. Phages have been used to introduce dCas9 systems to repress specific genes in the mouse gut microbiota (118). However, the temperate phage genome may need to be modified to inhibit lytic cycle activation, which could disrupt the maintenance of the introduced modifications (119).

Phagemids are plasmid elements packaged into phage capsids and delivered to the host. Phagemids are easily manipulated and, unlike temperate phages, do not carry phage replication genes, so no additional phages are produced post-infection. The commonly used phagemid M13 has been experimentally validated in mouse gut microbiota models, although it exhibits instability during *in vivo* experiments (113).

Phages may be directed to specific bacterial strains or to a wide range of strains. Although the phage cocktails used in clinical trials sometimes fail to encompass all biologically relevant strains (120), high-throughput phage screening can be used to ensure comprehensive strain coverage. The four-phage cocktail SNIPR001 was used to deliver CRISPR systems that depleted a wide array of phylogenetically diverse *E. coli* strains, paving the way for more precisely tailored phage therapies for specific medical conditions (120).

Although phages can modify complex bacterial communities *in situ* within the gut microbiota, most strategies necessitate both genetic modifications of the candidate phages and high-throughput screening to refine host range and specificity (121). Selective pressure on the targeted bacteria may result in mutated receptors, potentially leading to “immunity” to the phage. Moreover, the phages may be susceptible to conditions in the gut environment. Developing effective phage-based strategies requires characterizing and adapting the phages to ensure their stability and efficacy in the dynamic gut environment.

An alternative mode of *in situ* delivery of CRISPR systems to commensal gut bacteria is conjugation. Here, the bacterial type IV secretion system (T4SS) mediates the direct transfer of DNA from a donor cell to a nearby recipient cell (Fig. 3D). Conjugation has a broader range than phage-based methods but a lower transfer efficiency. Several conjugative plasmids, however, have relatively high transfer rates. For example, TP114, a plasmid originally discovered in the *Enterobacteriaceae* family, has demonstrated high conjugation rates in the gut of mouse models. This plasmid mobilized a CRISPR-Cas9 sequence targeting antibiotic-resistant strains of *E. coli* (Fig. 3E) and the pathogenic *Citrobacter rodentium*, depleting the murine microbiome of these specific strains (114).

In the DNA-editing all-in-one RNA-guided CRISPR-Cas transposase (DART) system, the conjugative plasmid VcDART is used to transfer an RNA-guided CRISPR-Cas transposase system for strain-specific *in situ* editing. This system allowed for strain-specific *in situ* editing of an infant fecal microbiota community by introducing an antibiotic-resistance marker gene at a strain-specific locus (Fig. 3F)**,** facilitating the isolation of the strain from a natural community (115). Additionally, MAGIC (metagenomic alteration of gut microbiome by *in situ* conjugation) and other strategies have demonstrated a high conjugation rate as well as a broad target cell range *in situ* during *in vivo* experiments using mouse models (122).

However, not all bacteria are amenable to existing conjugation mechanisms. Bacteria of the *Clostridia* class are highly prevalent in the human fecal microbiota (93), but as yet, very few conjugation techniques have been described for these organisms.

By modulating its target cell range and improving its *in situ* transfer efficiency, conjugation can be employed to knock down specific genes in specific bacteria within the gut microbiota and potentially could be adapted to deplete antibiotic-resistant bacterial populations. By designing CRISPR systems that target antibiotic resistance genes or by depleting pathogenic bacteria, conjugation mechanisms could play a pivotal role in addressing antibiotic resistance and pathogenic threats.

## FUTURE DIRECTIONS AND CONCLUSIONS

The application of CRISPR-based technologies to elucidate host-microbiome interactions is an exciting field of current research. Unlike traditional microbiological methods, CRISPR-based tools provide a robust mechanistic framework for characterizing the molecular mechanisms governing host-microbe interactions. These tools offer unprecedented precision and versatility for genetic manipulation and functional analysis, such as determining the contribution of specific genes to interactions within the microbiome, paving the way for novel insights and therapeutic strategies.

CRISPR technologies have diverse applications, including CRISPR-Cas9, base editing, and prime editing, for the precise gene editing of specific bacterial constituents within complex communities. CRISPRi/CRISPRa tools are being utilized to regulate gene expression, to elucidate gene function by high-throughput gene screening, and to design genetic circuits that can be used to develop whole-cell biosensors. CRISPR-based recorders can provide valuable insights into the dynamics of these complex ecosystems.

Several challenges persist for CRISPR technologies, particularly for their targeted delivery of these systems and the *in situ* editing of specific bacteria within complex environments. For example, the protective barriers of host organisms can block precise delivery. Nevertheless, recent breakthroughs, such as the delivery of a non-replicative base-editor system targeting *E. coli* in the mouse gut (Fig. 3C), demonstrate the potential for developing microbial therapies (113) and the feasibility of CRISPR-based interventions for modulating the gut microbiota. However, they also highlight the pressing need for more efficient delivery protocols and refined gene-editing techniques tailored to complex microbial ecosystems.

While *in vitro* assays utilizing host cell lines have illuminated some of the mechanisms underlying host-microbe interactions, such as host factors that affect susceptibility to infection, these findings require translation from *in vitro* models to *in vivo* scenarios. Organoid models and single-cell transcriptomics present promising avenues for studying host-microbe dynamics *in vivo* and acquiring a deeper comprehension of biological processes.

In conclusion, CRISPR-based tools provide powerful methods with which to unravel the intricate genetic and functional networks constituting the microbiome. New therapeutic strategies may be discovered by understanding the interplay between hosts and their microbial partners that these tools reveal.
